# Holistic screening of collapsing honey bee colonies in Spain: a case study

**DOI:** 10.1186/1756-0500-7-649

**Published:** 2014-09-15

**Authors:** Almudena Cepero, Jorgen Ravoet, Tamara Gómez-Moracho, José Luis Bernal, Maria J Del Nozal, Carolina Bartolomé, Xulio Maside, Aránzazu Meana, Amelia V González-Porto, Dirk C de Graaf, Raquel Martín-Hernández, Mariano Higes

**Affiliations:** Bee Pathology Laboratory, Consejería de Agricultura, Gobierno de Castilla-La Mancha, Centro Apícola Regional (CAR), Marchamalo, E-19180 Spain; Laboratory of Zoophysiology, Faculty of Sciences, Ghent University, Ghent, B-9000 Belgium; Grupo de Medicina Xenómica, CIMUS, Universidade de Santiago de Compostela, Santiago De Compostela, E-15782 Spain; Xenómica Comparada de Parásitos Humanos, IDIS, Santiago De Compostela, Spain; Analytical Chemistry Group. I.U.CINQUIMA, Universidad de Valladolid, Valladolid, E-47011 Spain; Departamento de Anatomía Patolóxica e Ciencias Forenses, Universidade de Santiago de Compostela, Santiago De Compostela, E-15782 Spain; Animal Health Department, Facultad de Veterinaria, Universidad Complutense de Madrid, Madrid, E-28040 Spain; Hive Products Laboratory, Consejería de Agricultura. Gobierno de Castilla-La Mancha, Centro Apícola Regional, Marchamalo, E-19180 Spain; Instituto de Recursos Humanos para la Ciencia y la Tecnología (INCRECYT-FEDER), Fundación Parque Científico y Tecnológico de Albacete, Albacete, Spain

**Keywords:** Honeybee, Colony collapse, Viruses, Parasites, Neonicotinoids, Palinology

## Abstract

**Background:**

Here we present a holistic screening of collapsing colonies from three professional apiaries in Spain. Colonies with typical honey bee depopulation symptoms were selected for multiple possible factors to reveal the causes of collapse.

**Results:**

Omnipresent were *Nosema ceranae* and Lake Sinai Virus. Moderate prevalences were found for Black Queen Cell Virus and trypanosomatids, whereas Deformed Wing Virus, Aphid Lethal Paralysis Virus strain Brookings and neogregarines were rarely detected. Other viruses, *Nosema apis*, *Acarapis woodi* and *Varroa destructor* were not detected. Palinologic study of pollen demonstrated that all colonies were foraging on wild vegetation. Consequently, the pesticide residue analysis was negative for neonicotinoids. The genetic analysis of trypanosomatids GAPDH gene, showed that there is a large genetic distance between *Crithidia mellificae* ATCC30254, an authenticated cell strain since 1974, and the rest of the presumed *C. mellificae* sequences obtained in our study or published. This means that the latter group corresponds to a highly differentiated taxon that should be renamed accordingly.

**Conclusion:**

The results of this study demonstrate that the drivers of colony collapse may differ between geographic regions with different environmental conditions, or with different beekeeping and agricultural practices. The role of other pathogens in colony collapse has to bee studied in future, especially trypanosomatids and neogregarines. Beside their pathological effect on honey bees, classification and taxonomy of these protozoan parasites should also be clarified.

**Electronic supplementary material:**

The online version of this article (doi:10.1186/1756-0500-7-649) contains supplementary material, which is available to authorized users.

## Background

The beekeeping sector is suffering unexpected losses in many countries to such extent that pollination services in some cases are jeopardized. Several explanatory ‘drivers’ are known: an increasing number of pathogens, invasive species, exposure to pesticides, reduced genetic diversity and some apicultural practices. The driver ‘pathogens’ has received much attention, so scientists have sought for years to find the dangerous mixture of transmittable bee diseases. They often had to face conflicting data and it became increasingly clear that the involved pathogens may vary significantly in different regions.

The early detection of Acute Bee Paralysis Virus (ABPV) [1], Chronic Bee Paralysis Virus (CBPV) [2] and Deformed Wing Virus (DWV) [3] in collapsing colonies has determined the experimental design of many subsequent health studies. Later on, the set of target viruses has increased: so, Kashmir Bee Virus (KBV), Black Queen Cell Virus (BQCV) and Sacbrood Virus (SBV) became commonly examined [4]. An unbiased microbiome study aimed at finding the cause of the Colony Collapse Disorder (CCD) in the USA has extended this ‘short list’ with the Israeli Acute Paralysis Virus (IAPV) and the microsporidian parasite *Nosema ceranae* [5]. Further investigations either indicate [6, 7] or confirmed [8–11] the important role of *N. ceranae* in temperate areas of the world. Very recently, the trypanosomatid *Crithidia mellificae* was found to be a contributory factor to the colony losses in Belgium [12].

The driver ‘invasive species’ refers mainly to the ectoparasitic mite *Varroa destructor.* With respect to bee mortality, the *Varroa*-load seems to be one of the few decisive factors who stand across the borders. In the USA, the small hive beetle (*Aethina tumida*) seems to be another leading cause of mortality in beekeeping operations [13].

Among proposed causes of bee mortality, the exposure of bees to pesticides received much attention lately and to such extent that the European Commission adopted a proposal [14] to restrict the use of 3 pesticides belonging to the neonicotinoids family (clothianidin, imidacloprid and thiametoxam) for a two years period. However, nationwide monitoring programs of honeybees’ exposure level to these crop protection products are rather scarce. Moreover, the real involvement of these pesticides is controversial [15].

Although there is a general agreement that the bee mortality problem is multifactorial [13, 16], monitoring programs or case studies that go beyond screening of bee pathogens are rather limited. Besides, the few nationwide studies or clinical studies focusing on pathogens in combination with pesticide residue analysis [17–19] are restricted to the current ‘short list’ of pathogens (DWV, ABPV, *V. destructor*).

Here we present a holistic screening (a case study) of collapsing honey bee colonies from three professional Spanish apiaries with high colony losses. Colonies with typical depopulation were analysed for the presence of multiple putative drivers of collapse: honey bee viruses, *Nosema* spp. *Varroa destructor*, *Acarapis woodi*, trypanosomatids, neogregarines, neonicotionoid insecticides and foraging flora. This study could also reveal what causal factors should be included in future Spanish monitoring programs [20–28].

## Results and discussion

The most widespread pathogens detected in the analyzed samples were *N. ceranae* and Lake Sinai Virus (LSV), found in all samples (100%, 10/10). Viruses of the ABPV complex, *Acarapis woodi, Nosema apis*, CBPV, SBV, SBPV and *Varroa destructor* were not detected (0%, 0/10) (Table 1).

**Table 1:** **Results of the honey bee pathogen (viruses, parasites) screening and pollen analyses (pesticide residue; palynology) on samples from collapsing colonies in Spain**

Sample information			Viruses				Parasites						Pesticide							Palinology	
Sample ID	Apiary	Location	BQCV	DWV	LSV	ALPV	*V. destructor*	*A. woodi*	*Trypanosomatids*	*Neogregarines*	*N. apis*	*N. ceranae*	Dinotefuran	Nitenpyram	Thiametoxan	Clothianidin	Imidacloprid	Acetamiprid	Thiacloprid	Plant genus	Source
55	1	Guadalajara	0	0	1	0	0	0	0	0	0	1	0	0	0	0	0	0	0	*Thymus*, *Raphanus*, *Rosmarinus*, *Salix*, *Brassica*, *Dorycnium*	Wild spp.
56	1	Guadalajara	0	0	1	0	0	0	0	0	0	1	0	0	0	0	0	0	0	*Thymus*, *Raphanus*, *Rosmarinus*, *Salix*, *Brassica*, *Dorycnium*	Wild spp.
57	1	Guadalajara	0	0	1	0	0	0	0	0	0	1	0	0	0	0	0	0	0	*Thymus*, *Raphanus*, *Rosmarinus*, *Salix*, *Brassica*, *Dorycnium*	Wild spp.
58	1	Guadalajara	1	0	1	0	0	0	0	0	0	1	0	0	0	0	0	0	0	*Thymus*, *Raphanus*, *Rosmarinus*, *Salix*, *Brassica*, *Dorycnium*	Wild spp.
59	1	Guadalajara	1	0	1	0	0	0	0	0	0	1	0	0	0	0	0	0	0	*Thymus*, *Raphanus*, *Rosmarinus*, *Salix*, *Brassica*, *Dorycnium*	Wild spp.
1980	2	Vizcaya	1	1	1	1	0	0	1	0	0	1	0	0	0	0	0	0	0	*Helianthemum*, *Raphanus*, *Rosmarinus*	Wild spp.
324	3	Murcia	1	0	1	0	0	0	1	1	0	1	0	0	0	0	0	0	0	*Thymus*, *Cistus*, *Salix*, *Lavandula*	Wild spp.
325	3	Murcia	1	0	1	1	0	0	1	0	0	1	0	0	0	0	0	0	0	*Thymus*, *Cistus*, *Salix*, *Lavandula*	Wild spp.
328	3	Murcia	0	0	1	0	0	0	1	0	0	1	0	0	0	0	0	0	0	*Thymus*, *Cistus*, *Salix*, *Lavandula*	Wild spp.
329	3	Murcia	0	0	1	0	0	0	0	0	0	1	0	0	0	0	0	0	0	*Thymus*, *Cistus*, *Salix*, *Lavandula*	Wild spp.

0 = not detected; 1 = detected.

The high prevalence (100%) of *N. ceranae* in the present study confirmed earlier reports [25, 29]. Its ability to evoke the collapse of honey bee colonies and to create the symptoms described by veterinarians (see methods), have been previously reported [10, 18, 20, 30–34]. *N. ceranae* plays a controversial role in the worldwide colony losses phenomenon [16]. While in Mediterranean areas a direct link between this pathogen and the honeybee losses has been reported [6, 8, 9, 11, 30, 35, 36], it can be excluded as main cause of losses in colder areas or continental climates [17, 37–39]. Nevertheless, in Belgium it was found that the adverse effects of this microsporidian can be enhanced in combination with the trypanosomatid *C. mellificae* [12]. In the present study, they were found in 4 samples and 2 apiaries. The three isolates studied (324, 325 and 1980) displayed several haplotypes, with variable levels of intra-isolate diversity (Table 2). Overall, *18S rDNA* was less variable (pooled total diversity *π* = 0.16 ± 0.06%; average ± SE) than *GAPDH* (*π* = 0.40 ± 0.14%) although the difference between both loci was not statistically significant. *C. mellificae* ATCC30254 also presented multiple haplotypes at the *GAPDH* locus, which reflects that this reference strain is not isogenic.

**Table 2:** **Estimates of Trypanosomatid diversity at all sites (** ***π*** **) expressed as percentage**

Isolate	*18S rDNA*		*GAPDH*	
	Average *π*	SE	Average *π*	SE
ATCC30254	0.00	0.00	0.68	0.23
324	0.06	0.06	0.86	0.35
325	0.30	0.11	0.29	0.11
1980	0.08	0.08	0.39	0.16
Pooled	0.16	0.06	0.40	0.14

*SE* standard error, calculated by a bootstrap procedure (3000 replicates) with MEGA5.

The pairwise comparison of the *18S rDNA* sequences revealed that *C. mellificae* ATCC30254 (KJ704242 – KJ704251) exhibited a single mutation that was not present either in the presumed *C. mellificae* sequences deposited in GenBank (KF607064.1 and AB745488.1) or in ours (KJ704218 – KJ707241). This single difference between *C. mellificae* ATCC30254 and the rest of the sequences is extremely important since any nucleotide variant in a highly conserved marker like the *18S rDNA* gene, which displays strong identity (about 99%) among different genus (Cepero et al., submitted), suggests that *C. mellificae* ATCC30254 and our sequences (which are mostly identical to KF607064.1 and AB745488.1; Additional file 1: Table S1) might represent genetically isolated organisms.

**Figure 1:**
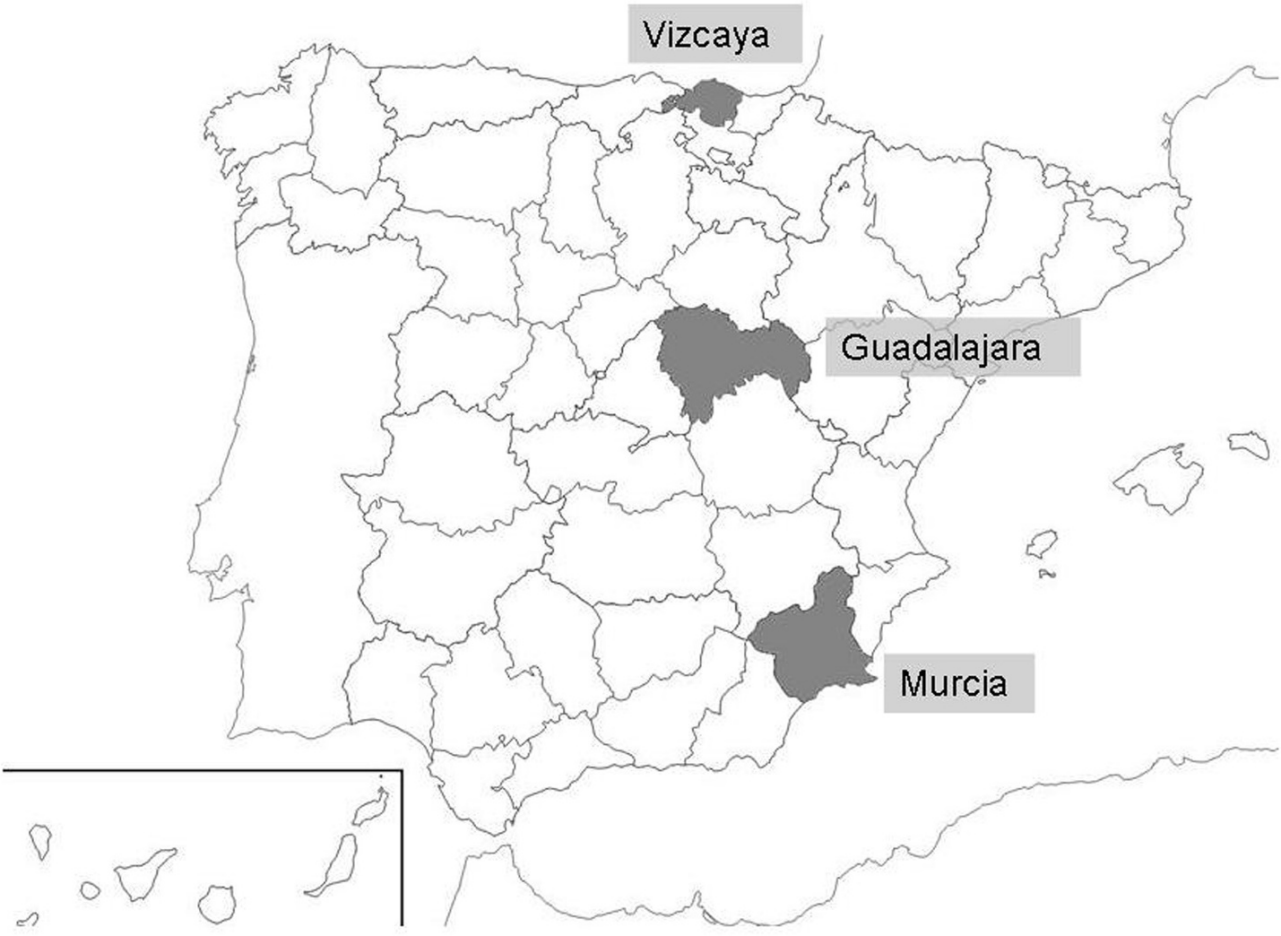
**Map of Spain.** Provinces of origin of the samples. In the north, Vizcaya. In the central area, Guadalajara. In the southeast, Murcia.

The analysis of the genetic distances between *GAPDH* sequences showed that *C. mellificae* ATCC30254 (KJ704273 – KJ704282) displayed a 6.89 ± 1.32% divergence with respect to the rest of the sequences, that included both the presumed *C. mellificae* sequences deposited in GenBank (AB716357.1, AB745489.1 and JF423199.1) and ours (KJ704252 – KJ704272), which, again, were in a large fraction identical to AB716357.1 and AB745489.1 (Additional file 2: Table S2). However, this finding is even more surprising if we bear in mind that total divergence estimates, as those calculated here, underestimate the neutral genetic distance between sequences (as replacement sites account for nearly 75% of the coding sequence and their evolutionary rate is severely limited by purifying selection). Consequently, to obtain more reliable estimates of divergence we performed pairwise comparisons at synonymous sites, which are considered neutral or nearly neutral. The outcome of this analysis is that there is a large genetic distance (23.47 ± 5.48%, Table 3) between *C. mellificae* ATCC30254, an authenticated cell strain since 1974, and the rest of the presumed *C. mellificae* sequences. This means that the latter group corresponds to a highly differentiated taxon that should be renamed accordingly. It is also worth noting the dispersal of *Crithidia* sp. sequences all over the tree (Figure 1), which questions their current taxonomic classification. In line with this, highly divergent *C. mellificae* lineages were previously mentioned [40].

**Table 3:** **Pairwise estimates of synonymous substitutions per synonymous site between** ***GAPDH*** **sequences**

	1	2	3	4	5	6	7	8	9	10	11	12	13	14
1														
2	0													
3	0	0												
4	1.58	1.58	1.58											
5	0	0	0	1.58										
6	1.60	1.60	1.60	0	1.60									
7	0	0	0	1.58	0	1.60								
8	1.58	1.58	1.58	0	1.58	0	1.58							
9	0	0	0	1.58	0	1.60	0	1.58						
10	1.60	1.60	1.60	0	1.60	0	1.60	0	1.60					
11	**24.66**	**24.66**	**24.66**	**26.77**	**24.66**	**26.30**	**24.66**	**26.77**	**24.66**	**26.30**				
12	**21.80**	**21.80**	**21.80**	**23.83**	**21.80**	**23.36**	**21.80**	**23.83**	**21.80**	**23.36**	3.23			
13	**23.21**	**23.21**	**23.21**	**25.29**	**23.21**	**24.81**	**23.21**	**25.29**	**23.21**	**24.81**	4.34	1.06		
14	**23.21**	**23.21**	**23.21**	**25.29**	**23.21**	**24.81**	**23.21**	**25.29**	**23.21**	**24.81**	1.06	2.14	3.23	

1: AB745489.1, 2: AB716357.1, 3: JF423199.1, 4: KJ704252 – 3, 5: KJ704254, 6: KJ704255, 7: KJ704256 – 63, 8: KJ704264, 9: KJ704265 – 69, KJ704271 – 72,10: KJ704270, 11: KJ704273, KJ704275, 12: KJ704274, KJ704281 – 82, 13: KJ704276 – 77, KJ704279, 14: KJ704278, KJ704280. Analyses were conducted in MEGA5 using the Nei-Gojobori model. All positions containing gaps and missing data were eliminated. Comparisons involving ATCC30254 and presumed *C. mellificae* sequences are highlighted in bold.

An unbiased metagenomic study in a Spanish non-professional apiary with collapsing colonies revealed the presence of several viruses, among which LSV complex and ALPV strain Brookings [41]. In this study, the former was present in all samples, and the latter in only a few (20%, 2/10). Sequencing of the LSV amplicons revealed that the strains from apiary 1 and 2 were almost identical (Genbank: KJ561228, KJ561229), but had a low resemblance to the strain from apiary 3 (Genbank: KJ561227). This strain has a high amino acid similarity (89%) with the Orf1 of LSV strain Navarra (Genbank: AGF84788). So, different LSV strains are present in Spain similar to the situation in Belgium [12] and the USA [40, 42]. The pathogenic implications of LSV in honey bee health status are under discussion. Cornman et al. [40] suggested a potential association between LSV and CCD colonies, although this observation was not found in a Belgian bee health screening [12]. In our work, LSV represents the most abundant virus in the analyzed samples collected during spring and summer. For these reason, the importance of LSV and the pathogenicity of different LSV strains should be further investigated.

Black Queen Cell Virus (BQCV) was found in 50% of samples (5/10) and in all apiaries. This result corresponds with those obtained before [24]. Aphid Lethal Paralysis Virus (ALPV) strain Brookings was found in 20% of samples (2/10) in two apiaries. The amino acid sequences were identical (>98%) to those detected previously in Belgium [12], Spain [41] and the USA [42] (Genbank: AGU62863, AGF84786, AEH26191). Curiously, Deformed Wing Virus (DWV) was only detected in one sample in one apiary, and this might be related to the low prevalence of *V. destructor* in the analyzed samples and therefore apiaries.

Neogregarines were detected in one sample. Direct sequencing indicated an infection by *Apicystis bombi*. However, the presence of overlapping peaks at particular points of the electropherograms suggested a potential mixture of templates, which was further investigated by cloning and sequencing. This process yielded sequences from another Apicomplexan parasite (99% identity with *Eimeriidae* or *Cryptosporidiidae* in Blastn), which was co-infecting the colony with *A. bombi* (whose presence was only confirmed by direct sequencing). Although *A. bombi* was thought to be mainly a bumblebee parasite [43, 44], there are increasing findings of that parasite in honey bees [45–47] since a molecular detection method became available [44]. Nevertheless, the amplification of other parasites with the same primers should be taken into account to avoid misdiagnosis in future studies.

Palinological analyses confirmed that the honeybees foraged on wild plants. As a consequence, no neonicotinoid residues in stored pollen were detected. Although these pesticides exhibited severe acute and sublethal effects on bees [48–50], their role as sole cause of colony loss is still not clear [13, 51]. Indeed, most published data have shown that acaricides, herbicides or other insecticide molecules, were more prevalent than neonicotinoids or phenilpirazoles in hives [22, 23, 52–55]. Our results confirm that neonicotinoids are not the only cause capable of causing the colony collapse of honey bee colonies.

## Conclusion

Many predictive markers and drivers have been suggested for honey bee colony collapses [5, 13, 56, 57]. The collapses of honey bee colonies in our study were not related with the presence of neonicotinoids or *V. destructor*. Instead, *N. ceranae* seems to be the main culprit of the colony losses in this study as already suggested in previous investigations [20, 30]. The role of other pathogens in colony collapse has to be studied in future, especially trypanosomatids and neogregarines. Beside their pathological effect on honey bees, classification and taxonomy of these protozoans also should be clarified. The results of this study clearly demonstrate that the drivers of colony collapse may differ between different geographic regions (see 16, 40).

## Methods

**Figure 2:**
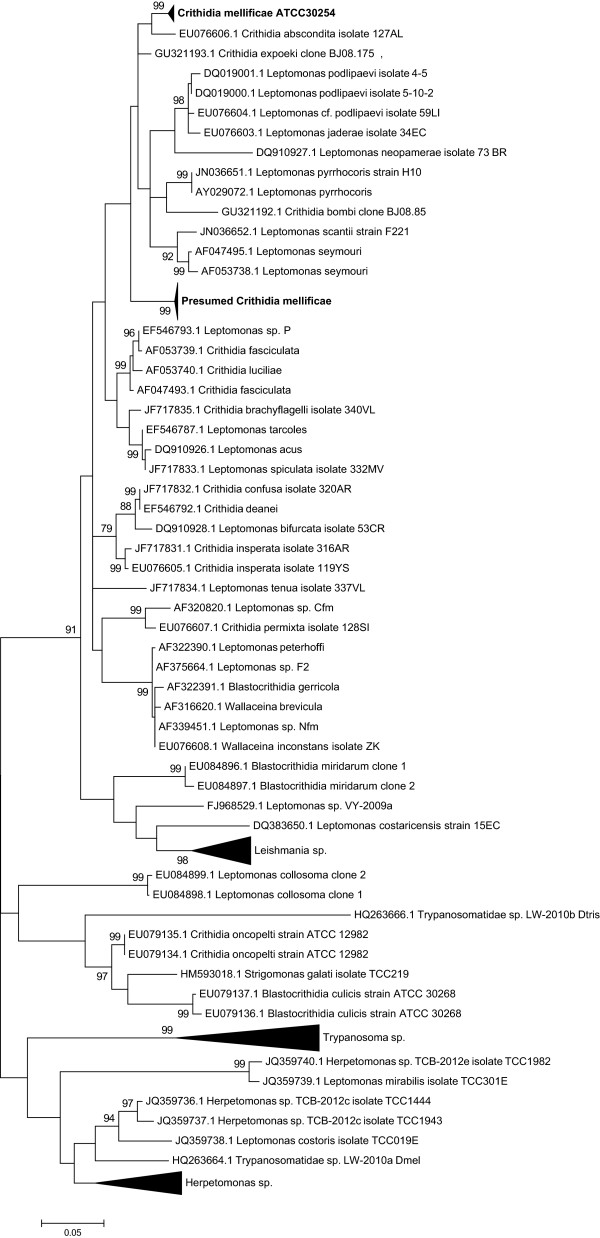
**Phylogenetic analysis of GAPDH sequences of trypanosomatids.** The tree was constructed using the Maximum Likelihood method (based on the General Time Reversible model, assuming a Gamma distribution and allowing some sites to be evolutionarily invariable). Bootstrap values (500 replicates) are shown next to the branches. Codon positions included were 1st + 2nd + 3rd + Noncoding. All positions containing gaps and missing data were eliminated. The clade of presumed *Crithidia mellificae* sequences, both from GenBank (AB745489.1, AB716357.1, JF423199.1) and from this work (KJ704252- KJ704272), was compressed for ease of visualisation and is displayed in bold. The same was done for *C. mellificae* ATCC30254 sequences (KJ704273 – KJ704282).

Samples were collected from three professional apiaries each located in a different region of Spain, all under different climatic and environmental conditions (Figure 2). The veterinarians responsible for these apiaries contacted the Centro Apícola Regional (CAR) pathology laboratory, because of alarming symptoms like depopulation and unusually high colony losses.

### Apiaries

Apiary 1: It is located in the Center of Spain in the province of Guadalajara and consisted of 400 bee colonies wintered in 2012, distributed in five apiaries all sited in the same province. In early spring 2013, 70% of the colonies had collapsed, with clear symptoms of depopulation such as disappearance of adult bees and unattended brood. Chalkbrood and foulbrood were not reported by the responsible veterinarian. The 30% of surviving colonies in early spring had a small adult bee population and low vitality. To perform the analysis, 25 weak surviving bee colonies were sampled (worker bees and pollen) in August 2013.

Apiary 2 2: It is located in the province of Vizcaya, Northern Spain. In winter 2012, it had 300 bee colonies distributed in 3 apiaries very closed each other. In early spring 2013, all colonies showed clear signs of depopulation and imminent risk of collapse. The responsible veterinarian has not reported other disease symptoms, although the bee colonies showed a very marked lack of vitality. For analysis, ten collapsing colonies were sampled (worker bees and pollen) in August 2013.

Apiary 3: It is located in the Southeast of Spain, in the province of Murcia. This apiary wintered 175 bee colonies distributed in 4 apiaries in 2011 and the 90% of them had collapsed in early spring 2012. For the analysis, 16 weak surviving colonies were sampled (worker bees and pollen) in May 2012.

### Honey bee sampling

Samples of worker honey bees (n = 100 worker bees) and stored pollen, were collected from the brood chamber by the veterinarians in charge and sent to the CAR bee Pathology Laboratory in spring (samples from Vizcaya and Murcia) o early summer (samples from Guadalajara). Samples from the apiary 1 (n = 25; five per apiary) were pooled together to obtain five pooled samples of five colonies each, one pool sample per apiary. Samples from apiary 2 (n = 10) were pooled into a single sample. Samples for apiary 3 (n = 16; four per apiary) were processed as the first apiary so there were four pooled samples from four colonies each (one pooled sample per apiary).

### *Varroa destructor*detection

The presence of *V. destructor* in worker honey bee samples were analyzed as described previously [19, 20].

### DNA and RNA extraction

All bee samples were macerated in AL buffer 50% (Qiagen) as previously described [24] using sterile bags with filter. Resulting pellets were used for DNA extraction and supernatants for RNA extraction. They were frozen at −80°C until extraction of the nucleic acids.

For DNA extraction, the pellet was resuspended in 3 ml sterile water and 400 μl was transferred into a 96-well plate (Qiagen) with glass beads (2 mm diameter, Sigma). Samples were then processed as previously described [25]. Briefly, the plates were shaken for 6 min at 30 Hz. Afterwards, 150 μL of each sample was transferred to a Deepwell plate (Eppendorf) with 30 μl of ATL buffer (Qiagen) and 20 μl of Proteinase K (Qiagen). After overnight incubation at 56°C, DNA was extracted using the BS96 DNA Tissue extraction protocol in a BioSprint machine (Qiagen). Plates were stored at −20°C until use.

RNA was extracted from the supernatant using the DNeasy Blood & Tissue kit (Qiagen), according to the manufacturer’s instructions for RNA isolation. Briefly, 200 μl PBS and 1 μl carrier RNA were added to 220 μl of the resulting supernatants. After 10 min incubation at 56°C, 230 μl ethanol was added. Subsequent to binding and washing, RNA elution was accomplished with 50 μl nuclease-free water.

The *C. mellificae* reference strain ATCC30254 was included in the analysis for sequence comparison with field isolates. This strain was first cultivated as recommended in ATCC medium 355. Further sub-cultivation was performed in Brain Heart Infusion (BHI) medium as described by Popp and Lattorf [58]. Visible and isolated colonies were taken and resuspended in milliQ water (PCR-quality) and the processed for DNA extraction as above described.

### PCR and MLPA analysis

A broad pathogen screening of the honeybee samples was performed using published PCR assays for *Acarapis* spp. mites [27], *Nosema apis* and *N. ceranae* [25], trypanosomatids and neogregarines [44]. Given that the *18S rDNA* gene alone is unsuitable to classify trypanosomatid/s infecting honeybee colonies (Cepero et al., submitted), these were also amplified at the *GAPDH* locus using CrGD-1 F (5′ GGTCGCCGTGGTGGAC 3′) and CrGD-1R (5′ CGTCGCCGTGTAGGAGTGA 3′). Since these oligos did not produce an amplicon from ATCC30254, this strain was PCR-amplified with Tryp-1 F (5′ CCGAGTACTTCKCSTACCAG 3′) and Tryp-1R (5′ AGCCGAGGATGCCCTTCAT 3′), a set of primers that should amplify most *Crithidia* and *Leptomonas* species published in GenBank to date. As template, we used 5 μl DNA in each reaction.

For virus analysis, the BeeDoctor test, a ‘multiplex-ligation probe dependent amplification’ (MLPA) based method capable of detecting CBPV, DWV-complex, ABPV-complex, BQCV, SBPV and SBV, was performed as described before [59], starting from 3 μl RNA. The amplified MLPA products were analyzed using 4% high resolution agarose gel electrophoresis.

In addition, we performed additional RT-PCR analyses for few viruses. Using random hexamer primers, 500 ng RNA was retro-transcribed with the RevertAid H Minus First Strand cDNA Synthesis Kit (Thermo Scientific). For ALPV strain Brookings and LSV complex detection, we used 1 μl cDNA template in the PCR test described by Runckel et al., [42] and Ravoet et al. [12] respectively.

### Sequencing and cloning

Positive samples of ALPV strain Brookings, LSV complex, trypanosomatids and neogregarines were re-analyzed using the HotStar HiFidelity Polymerase Kit (Qiagen) (viruses) or the Expand High Fidelity^PLUS^ PCR System (Roche Diagnostics) (protozoans).

Protozoan amplicons were cloned using the Topo TA Cloning Kit (Invitrogen) after purification with the QIAquick PCR Purification Kit (Qiagen). Plasmids were purified using the QIAprep Spin Miniprep Kit (QIAgen). Both strands were sequenced with M13 primers on an automated ABI3730XL sequencer using Big Dye (Applied Biosystems). ALPV strain Brookings and LSV complex amplicons were bidirectional sequenced on an ABI3130XL with gene-specific primers.

Sequences were checked for accurate base calling using CodonCode Aligner (CodonCode Corporation) and their identity verified by means of a nucleotide BLAST. Alignments were manually edited with BioEdit [60] and submitted to GenBank (KJ704218 – KJ704282).

Estimates of overall and pairwise nucleotide diversity were obtained with MEGA v.5.05 [61]. These were measured as *π*, applying the Jukes and Cantor (JC) correction [62], and their standard error (SE) calculated by a bootstrap procedure (3000 replicates). This software was also used to construct a maximum likelihood phylogeny under the GTR + G + I model (General Time Reversible + Gamma distributed + Invariable sites) that was selected as best model by applying the Akaike information criterion (AIC), as implemented in MEGA*.* The reliability of the tree topology was tested by bootstrap support (500 replicates).

### Pollen sampling

In order to obtain a representative pollen samples from each colony, stored pollen (3 squares of approximately 10 × 10 cm each) collected from the brood chamber combs were extracted aseptically from five colonies of each apiary [20, 22]. Each pollen sample was divided in two aliquots. One aliquot of 100 g was used for neonicotinoid screening, while the other aliquot of 5–10 g was used for palinological analysis. Samples were stored at −20°C until further use.

### Neonicotinoids analysis

Seven neonicotinoid insecticides (acetamiprid, clothianidin, dinotefuran, imidacloprid, nitenpyram, thiacloprid, and thiamethoxam) were analyzed pollen with a previous methodology developed by Yáñez et al. [63]. It should be mentioned that the equipment, methods and reagents were the same than the described in this previous research. The sample treatment consisted of a solid–liquid extraction of the neonicotinoids from pollen with dichloromethane, followed by evaporation and reconstitution. Briefly, 2 g pollen sample and 10 mL of dichloromethane were transferred to a centrifuge tube. The mixture was mechanically shaken for 10 min at 800 oscillations per minute in a Vibromatic and then centrifuged for 10 min at 25°C and 10,400 × g. Following this, the supernatant was collected, filtered through paper filter, transferred to a 25-mL conical flask, and then evaporated until dry in a rotary evaporator at 40°C. The dry extract was reconstituted with 1 mL of a water and acetonitrile mixture (50:50, v/v) and the resulting solution was passed through a syringe filter, after which a 15-μL aliquot was injected into a liquid chromatograph (LC) coupled to an electrospray ionization mass spectrometry detector (ESI-MS). Once the neonicotinoids were extracted, they were determined using an optimized LC-ESI-MS method, which was validated in terms of selectivity, linearity, precision and recovery. The limits of detection (LOD) and quantification (LOQ) were 0.4–2.8 μg/kg and 1.2–9.1 μg/kg, respectively, and the extraction recoveries were between 86 and 106% in all cases.

### Palinological analysis

The palinological analysis was performed as described previously [20, 22]. Briefly, pollen grains were isolated from each sample and cleaned up by the Erdtman method [64]. Species identification was performed using a photographic atlas [65, 66] and the pollen slides reference collection at the CAR. Briefly, pollen were extracted by diluting 0.5 g in 10 ml of acidulated water (0.5% sulphuric acid) and then centrifuged at 2,500 rpm for 15 min. The pellet was washed with double-distilled (dd) water, centrifuged and resuspended in ddH2O. 200 μl of this suspension was placed onto a glycerin jelly slide and examined microscopically in order to identify the pollen.

## Ethics statement

In Europe, the EU Directive 2010/63/EU on the protection of animals used for scientific purposes laid the down the ethical framework for the use of animals in scientific experiments. The scope of this directive also includes specific invertebrate species, i.e. cephalopods, but no insects. Thus, according to European legislation no specific permits were required for the described studies.

## Electronic supplementary material

Additional file 1: Table S1: Pairwise estimates of evolutionary divergence between *18S rDNA* sequences (expressed as %). (DOC 176 KB)

Additional file 2: Table S2: Pairwise estimates of evolutionary divergence between *GAPDH* sequences (expressed as %). (DOC 190 KB)

## References

[1] Ball BV, Allen MF (1988). The prevalence of pathogens in honey bee (*apis mellifera*) colonies infested with the parasitic mite *varroa jacobsoni*. Ann Appl Biol.

[2] Faucon JP, Mathieu L, Ribiere M, Martel CA, Drajnudel P, Zeggane S, Aurieres C, Aubert MFA (2002). Honey bee winter mortality in France in 1999 and 2000. Bee world.

[3] Nordström S, Fries I, Aarhus A, Hansen H, Korpela S (1999). Virus infections in nordic honey bee colonies with no, low or severe *varroa jacobsoni* infestations. Apidologie.

[4] Tentcheva D, Gauthier L, Zappulla N, Dainat B, Cousserans F, Colin ME, Bergoin M (2004). Prevalence and seasonal variations of six bee viruses in *apis mellifera L.* and *varroa destructor* mite populations in France. Appl Environ Microbiol.

[5] Cox-Foster DL, Conlan S, Holmes EC, Palacios G, Evans JD, Moran NA, Quan P-L, Briese T, Hornig M, Geiser DM, Martinson V, VanEngelsdorp D, Kalkstein AL, Drysdale A, Hui J, Zhai J, Cui L, Hutchison SK, Simons JF, Egholm M, Pettis JS, Lipkin WI (2007). A metagenomic survey of microbes in honey bee colony collapse disorder. Science.

[6] Higes M, Martín-Hernández R, Meana A (2006). *Nosema ceranae*, a new microsporidian parasite in honeybees in Europe. J Invertebr Pathol.

[7] Martín-Hernández R, Meana A, Prieto L, Salvador AM, Garrido-Bailón E, Higes M (2007). Outcome of colonization of *Apis mellifera* by *Nosema ceranae*. Appl Environ Microbiol.

[8] Hatjina F, Tsoktouridis G, Bouga M, Charistos L, Evangelou V, Avtzis D, Meeus I, Brunain M, Smagghe G, de Graaf DC (2011). Polar tube protein gene diversity among *Nosema ceranae* strains derived from a Greek honey bee health study. J Invertebr Pathol.

[9] Soroker V, Hetzroni A, Yakobson B, David D, David A, Voet H, Slabezki Y, Efrat H, Levski S, Kamer Y, Klinberg E, Zioni N, Inbar S, Chejanovsky N (2011). Evaluation of colony losses in Israel in relation to the incidence of pathogens and pests. Apidologie.

[10] Villa JD, Bourgeois AL, Danka RG (2013). Negative evidence for effects of genetic origin of bees on *Nosema ceranae*, positive evidence for effects of *Nosema ceranae* on bees. Apidologie.

[11] Lodesani M, Costa C, Besana A, Dall’Olio R, Franceschetti S, Tesoriero D, Vaccari G (2014). Impact of control strategies for *Varroa destructor* on colony survival and health in northern and central regions of Italy. J Apic Res.

[12] Ravoet J, Maharramov J, Meeus I, De Smet L, Wenseleers T, Smagghe G, de Graaf DC (2013). Comprehensive bee pathogen screening in Belgium reveals *Crithidia mellificae* as a new contributory factor to winter mortality. PLoS One.

[13] VanEngelsdorp D, Meixner MD (2010). A historical review of managed honey bee populations in Europe and the United States and the factors that may affect them. J Invertebr Pathol.

[14] **Commission implementing regulation (EU) No 485/2013 of 24 May 2013 amending implementing regulation (EU) No 540/2011**. http://eur-lex.europa.eu/LexUriServ/LexUriServ.do?uri=OJ:L:2013:139:0012:0026:EN:PDF, Accessed 12 May 2014

[15] Staveley JP, Law SA, Fairbrother A, Menzie CA (2014). A causal analysis of observed declines in managed honey bees (*Apis mellifera*). Hum Ecol Risk Assess.

[16] Higes M, Meana A, Bartolomé C, Botías C, Martín-Hernández R (2013). *Nosema ceranae* (microsporidia), a controversial 21st century honey bee pathogen. Environ Microbiol Rep.

[17] Gisder S, Hedtke K, Möckel N, Frielitz M-C, Linde A, Genersch E (2010). Five-year cohort study of *Nosema spp*. in Germany: does climate shape virulence and assertiveness of *Nosema ceranae*?. Appl Environ Microbiol.

[18] Higes M, Martín-Hernández R, Martínez-Salvador A, Garrido-Bailón E, González-Porto AV, Meana A, Bernal JL, Del Nozal MJ, Bernal J (2010). A preliminary study of the epidemiological factors related to honey bee colony loss in Spain. Environ Microbiol Rep.

[19] Garrido-Bailón E (2012). PhD Thesis. Repercusión Potencial en la Cabaña Apícola Española de Agentes Nosógenos Detectados en Colonias de Apis Mellifera Iberiensis.

[20] Higes M, Martín-Hernández R, Garrido-Bailón E, González-Porto AV, García-Palencia P, Meana A, Del Nozal MJ, Mayo R, Bernal JL (2009). Honeybee colony collapse due to *nosema ceranae* in professional apiaries. Environ Microbiol Rep.

[21] Higes M, Martín-Hernández R, García-Palencia P, Marín P, Meana A (2009). Horizontal transmission of *nosema ceranae* (microsporidia) from worker honeybees to queens (*apis mellifera*). Environ Microbiol Rep.

[22] Bernal J, Garrido-Bailón E, Del Nozal MJ, González-Porto AV, Martín-Hernández R, Diego JC, Jiménez JJ, Bernal JL, Higes M (2010). Overview of pesticide residues in stored pollen and their potential effect on bee colony (*Apis mellifera*) losses in Spain. J Econ Entomol.

[23] Bernal J, Martín-Hernández R, Diego JC, Nozal MJ, Gozalez-Porto AV, Bernal JL, Higes M (2011). An exposure study to assess the potential impact of fipronil in treated sunflower seeds on honey bee colony losses in Spain. Pest Manag Sci.

[24] Antúnez K, Anido M, Garrido-Bailón E, Botías C, Zunino P, Martínez-Salvador A, Martín-Hernández R, Higes M (2012). Low prevalence of honeybee viruses in Spain during 2006 and 2007. Res Vet Sci.

[25] Martín-Hernández R, Botías C, Bailón EG, Martínez-Salvador A, Prieto L, Meana A, Higes M (2012). Microsporidia infecting *apis mellifera*: coexistence or competition. Is *nosema ceranae* replacing *nosema apis*?. Environ Microbiol.

[26] Garrido-Bailón E, Martín-Hernández R, Bernal J, Bernal JL, Martínez-Salvador A, Barrios L, Meana A, Higes M (2010). Short communication. The detection of Israeli acute paralysis virus (IAPV), fipronil and imidacloprid in professional apiaries are not related with massive honey bee colony loss in Spain. Spanish J Agric Res.

[27] Garrido-Bailón E, Bartolomé C, Prieto L, Botías C, Martínez-Salvador A, Meana A, Martín-Hernández R, Higes M (2012). The prevalence of *acarapis woodi* in Spanish honey bee (*apis mellifera*) colonies. Exp Parasitol.

[28] Garrido-Bailón E, Higes M, Martínez-Salvador A, Antúnez K, Botías C, Meana A, Prieto L, Martín-Hernández R (2013). The prevalence of the honeybee brood pathogens *ascosphaera apis*, *paenibacillus larvae* and *melissococcus plutonius* in Spanish apiaries determined with a new multiplex PCR assay. Microb Biotechnol.

[29] Botías C, Martín-Hernández R, Barrios L, Garrido-Bailón E, Nanetti A, Meana A, Higes M (2012). *Nosema spp*. parasitization decreases the effectiveness of acaricide strips (Apivar(®)) in treating varroosis of honey bee (*Apis mellifera iberiensis*) colonies. Environ Microbiol Rep.

[30] Higes M, Martín-Hernández R, Botías C, Garrido-Bailón E, González-Porto AV, Barrios L, Del Nozal MJ, Bernal JL, Jiménez JJ, García-Palencia P, Meana A (2008). How natural infection by *Nosema ceranae* causes honeybee colony collapse. Environ Microbiol.

[31] Higes M, Martín-Hernández R, Meana A (2010). *Nosema ceranae* in Europe: an emergent type C nosemosis. Apidologie.

[32] Botías C, Martín-Hernández R, Días J, García-Palencia P, Matabuena M, Juarranz A, Barrios L, Meana A, Nanetti A, Higes M (2012). The effect of induced queen replacement on *Nosema spp.* infection in honey bee (*Apis mellifera iberiensis*) colonies. Environ Microbiol.

[33] Botías C, Martín-Hernández R, Barrios L, Meana A, Higes M (2013). *Nosema spp.* infection and its negative effects on honey bees (*Apis mellifera iberiensis*) at the colony level. Vet Res.

[34] Goblirsch M, Huang ZY, Spivak M (2013). Physiological and behavioral changes in honey bees (*Apis mellifera*) induced by *Nosema ceranae* infection. PLoS One.

[35] Borneck R, Viry A, Martín-Hernández R, Higes M (2010). Honey bee colony losses in the Jura region, France and related pathogens. J Apic Res.

[36] Bacandritsos N, Granato A, Budge G, Papanastasiou I, Roinioti E, Caldon M, Falcaro C, Gallina A, Mutinelli F (2010). Sudden deaths and colony population decline in Greek honey bee colonies. J Invertebr Pathol.

[37] Stevanovic J, Stanimirovic Z, Genersch E, Kovacevic SR, Ljubenkovic J, Radakovic M, Aleksic N (2011). Dominance of *nosema ceranae* in honey bees in the Balkan countries in the absence of symptoms of colony collapse disorder. Apidologie.

[38] Stevanovic J, Simeunovic P, Gajic B, Lakic N, Radovic D, Fries I, Stanimirovic Z (2013). Characteristics of *Nosema ceranae* infection in Serbian honey bee colonies. Apidologie.

[39] Hedtke K, Jensen PM, Jensen AB, Genersch E (2011). Evidence for emerging parasites and pathogens influencing outbreaks of stress-related diseases like chalkbrood. J Invertebr Pathol.

[40] Cornman RS, Tarpy DR, Chen Y, Jeffreys L, Lopez D, Pettis JS, VanEngelsdorp D, Evans JD (2012). Pathogen webs in collapsing honey bee colonies. PLoS One.

[41] Granberg F, Vicente-Rubiano M, Rubio-Guerri C, Karlsson OE, Kukielka D, Belák S, Sánchez-Vizcaíno JM (2013). Metagenomic detection of viral pathogens in Spanish honeybees: co-infection by aphid lethal paralysis, israel acute paralysis and lake sinai viruses. PLoS One.

[42] Runckel C, Flenniken ML, Engel JC, Ruby JG, Ganem D, Andino R, DeRisi JL (2011). Temporal analysis of the honey bee microbiome reveals four novel viruses and seasonal prevalence of known viruses, *Nosema*, and *Crithidia*. PLoS One.

[43] Rutrecht ST, Brown MJF (2008). The life-history impact and implications of multiple parasites for bumble bee queens. Int J Parasitol.

[44] Meeus I, de Graaf DC, Jans K, Smagghe G (2010). Multiplex PCR detection of slowly-evolving trypanosomatids and neogregarines in bumblebees using broad-range primers. J Appl Microbiol.

[45] Plischuk S, Meeus I, Smagghe G, Lange CE (2011). *Apicystis bombi* (apicomplexa: neogregarinorida) parasitizing *apis mellifera* and *bombus terrestris* (hymenoptera: apidae) in Argentina. Environ Microbiol Rep.

[46] Maharramov J, Meeus I, Maebe K, Arbetman M, Morales C, Graystock P, Hughes WOH, Plischuk S, Lange CE, de Graaf DC, Zapata N, de La Rosa JJ P, Murray TE, Brown MJF, Smagghe G (2013). Genetic variability of the neogregarine *Apicystis bombi*, an etiological agent of an emergent bumblebee disease. PLoS One.

[47] Morimoto T, Kojima Y, Yoshiyama M, Kimura K, Yang B, Peng G, Kadowaki T (2013). Molecular detection of protozoan parasites infecting Apis mellifera colonies in Japan. Environ Microbiol Rep.

[48] Desneux N, Decourtye A, Delpuech JM (2007). The sublethal effects of pesticides on beneficial arthropods. Annu Rev Entomol.

[49] Belzunces LP, Tchamitchian S, Brunet J-L (2012). Neural effects of insecticides in the honey bee. Apidologie.

[50] Henry M, Béguin M, Requier F, Rollin O, Odoux J-F, Aupinel P, Aptel J, Tchamitchian S, Decourtye A (2012). A common pesticide decreases foraging success and survival in honey bees. Science.

[51] VanEngelsdorp D, Evans JD, Saegerman C, Mullin C, Haubruge E, Nguyen BK, Frazier M, Frazier J, Cox-Foster D, Chen Y, Underwood R, Tarpy DR, Pettis JS (2009). Colony collapse disorder: a descriptive study. PLoS One.

[52] Johnson RM, Ellis MD, Mullin CA, Frazier M (2010). Pesticides and honey bee toxicity – USA. Apidologie.

[53] Mullin CA, Frazier M, Frazier JL, Ashcraft S, Simonds R, VanEngelsdorp D, Pettis JS (2010). High levels of miticides and agrochemicals in North American apiaries: implications for honey bee health. PLoS One.

[54] Lambert O, Piroux M, Puyo S, Thorin C, L’Hostis M, Wiest L, Buleté A, Delbac F, Pouliquen H (2013). Widespread occurrence of chemical residues in beehive matrices from apiaries located in different landscapes of Western France. PLoS One.

[55] Pettis JS, Lichtenberg EM, Andree M, Stitzinger J, Rose R, VanEngelsdorp D (2013). Crop pollination exposes honey bees to pesticides which alters their susceptibility to the gut pathogen *Nosema ceranae*. PLoS One.

[56] Dainat B, Evans JD, Chen YP, Gauthier L, Neumann P (2012). Predictive markers of honey bee colony collapse. PLoS One.

[57] Farooqui T (2013). A potential link among biogenic amines-based pesticides, learning and memory, and colony collapse disorder: a unique hypothesis. Neurochem Int.

[58] Popp M, Lattorff HMG (2011). A quantitative *in vitro* cultivation technique to determine cell number and growth rates in strains of crithidia bombi (trypanosomatidae), a parasite of bumblebees. J Eukaryot Microbiol.

[59] De Smet L, Ravoet J, de Miranda JR, Wenseleers T, Mueller MY, Moritz RFA, de Graaf DC (2012). BeeDoctor, a versatile MLPA-based diagnostic tool for screening bee viruses. PLoS One.

[60] Hall TA (1999). BioEdit: a user-friendly biological sequence alignment editor and analysis program for Windows 95/98/NT. Nucleic Acids Symp Ser.

[61] Tamura K, Peterson D, Peterson N, Stecher G, Nei M, Kumar S (2011). MEGA5: molecular evolutionary genetics analysis using maximum likelihood, evolutionary distance, and maximum parsimony methods. Mol Biol Evol.

[62] Jukes TH, Cantor CR, Munro HN (1969). Evolution of Protein Molecules. Mammalian Protein Metabolism.

[63] Yáñez KP, Martín MT, Bernal JL, Nozal MJ, Bernal J (2014). Trace analysis of seven neonicotinoid insecticides in bee pollen by solid–liquid extraction and liquid chromatography coupled to electrospray ionization mass spectrometry. Food Anal Methods.

[64] Erdtman G (1969). Handbook of palynology, morphology, taxonomy, ecology: an introduction to the study of pollen grains and spores.

[65] Valdés B (1987). Atlas Polínico de Andalucía Occidental.

[66] Faegri K, Iversen J (1989). Textbook of Pollen Analysis.

