# Supplementary material for: Holistic screening of collapsing honey bee colonies in Spain: a case study
Source: BMC Res Notes. 2014 Sep 15;7:649. doi: 10.1186/1756-0500-7-649 (PMC4180541; doi:10.1186/1756-0500-7-649)
Supplement: Supplementary file 1 — Additional file 1: Table S1: Pairwise estimates of evolutionary divergence between 18S rDNA sequences (expressed as %). (DOC 176 KB) [file 13104_2014_3189_MOESM1_ESM.doc]

**Additional file 1: Table S1**. **Pairwise estimates of evolutionary divergence between *18S rDNA* sequences (expressed as %).**

|  |  | 1 | 2 | 3 | 4 | 5 | 6 | 7 | 8 | 9 | 10 |
| --- | --- | --- | --- | --- | --- | --- | --- | --- | --- | --- | --- |
| 1 |  |  |  |  |  |  |  |  |  |  |  |
| 2 |  | 0.00 |  |  |  |  |  |  |  |  |  |
| 3 |  | 0.26 | 0.26 |  |  |  |  |  |  |  |  |
| 4 |  | 0.00 | 0.00 | 0.26 |  |  |  |  |  |  |  |
| 5 |  | 0.26 | 0.26 | 0.52 | 0.26 |  |  |  |  |  |  |
| 6 |  | 0.00 | 0.00 | 0.26 | 0.00 | 0.26 |  |  |  |  |  |
| 7 |  | 0.52 | 0.52 | 0.78 | 0.52 | 0.78 | 0.52 |  |  |  |  |
| 8 |  | 0.26 | 0.26 | 0.52 | 0.26 | 0.52 | 0.26 | 0.78 |  |  |  |
| 9 |  | 0.00 | 0.00 | 0.26 | 0.00 | 0.26 | 0.00 | 0.52 | 0.26 |  |  |
| 10 |  | 0.26 | 0.26 | 0.52 | 0.26 | 0.52 | 0.26 | 0.78 | 0.52 | 0.26 |  |

1: KF607064.1, 2: AB745488.1, 3: KJ704242 – 51, 4: KJ704218 – 24, 5: KJ704225, 6: KJ704226 – 29, KJ704233, 7: KJ704230, 8: KJ704231 – 32, KJ704234 – 35, 9: KJ704236, KJ704238 – 41, 10: KJ704237. Analyses were conducted in MEGA5 using the Jukes-Cantor model. All positions containing gaps and missing data were eliminated. Comparisons between ATCC30254 and presumed *C. mellificae* sequences are highlighted in grey.
