# Supplementary material for: Holistic screening of collapsing honey bee colonies in Spain: a case study
Source: BMC Res Notes. 2014 Sep 15;7:649. doi: 10.1186/1756-0500-7-649 (PMC4180541; doi:10.1186/1756-0500-7-649)
Supplement: Supplementary file 2 — Additional file 2: Table S2: Pairwise estimates of evolutionary divergence between GAPDH sequences (expressed as %). (DOC 190 KB) [file 13104_2014_3189_MOESM2_ESM.doc]

**Additional file 2: Table S2**. **Pairwise estimates of evolutionary divergence between *GAPDH* sequences (expressed as %).**

|  |  | 1 | 2 | 3 | 4 | 5 | 6 | 7 | 8 | 9 | 10 | 11 | 12 | 13 | 14 |
| --- | --- | --- | --- | --- | --- | --- | --- | --- | --- | --- | --- | --- | --- | --- | --- |
| 1 |  |  |  |  |  |  |  |  |  |  |  |  |  |  |  |
| 2 |  | 0 |  |  |  |  |  |  |  |  |  |  |  |  |  |
| 3 |  | 0.27 | 0.27 |  |  |  |  |  |  |  |  |  |  |  |  |
| 4 |  | 1.07 | 1.07 | 1.34 |  |  |  |  |  |  |  |  |  |  |  |
| 5 |  | 0.27 | 0.27 | 0.53 | 1.34 |  |  |  |  |  |  |  |  |  |  |
| 6 |  | 1.07 | 1.07 | 1.34 | 0.53 | 1.34 |  |  |  |  |  |  |  |  |  |
| 7 |  | 0 | 0 | 0.27 | 1.07 | 0.27 | 1.07 |  |  |  |  |  |  |  |  |
| 8 |  | 0.27 | 0.27 | 0.53 | 1.34 | 0.53 | 1.34 | 0.27 |  |  |  |  |  |  |  |
| 9 |  | 1.07 | 1.07 | 1.34 | 0.53 | 1.34 | 0.53 | 1.07 | 1.34 |  |  |  |  |  |  |
| 10 |  | 0 | 0 | 0.27 | 1.07 | 0.27 | 1.07 | 0 | 0.27 | 1.07 |  |  |  |  |  |
| 11 |  | 6.94 | 6.94 | 6.65 | 8.12 | 7.23 | 8.12 | 6.94 | 7.23 | 8.12 | 6.94 |  |  |  |  |
| 12 |  | 6.36 | 6.36 | 6.08 | 7.53 | 6.65 | 7.53 | 6.36 | 6.65 | 7.53 | 6.36 | 0.80 |  |  |  |
| 13 |  | 6.65 | 6.65 | 6.36 | 7.82 | 6.94 | 7.82 | 6.65 | 6.94 | 7.82 | 6.65 | 1.07 | 0.27 |  |  |
| 14 |  | 6.65 | 6.65 | 6.36 | 7.82 | 6.94 | 7.82 | 6.65 | 6.94 | 7.82 | 6.65 | 0.27 | 0.53 | 0.80 |  |

1: AB745489.1, 2: AB716357.1, 3: JF423199.1, 4: KJ704252 -53, 5: KJ704254, 6: KJ704255, 7: KJ704256 – 57, KJ704259 – 61, KJ704263, 8: KJ704258, KJ704262, 9: KJ704264, KJ704270, 10: KJ704265 – 69, KJ704271 -72, 11: KJ704273, KJ704275, 12: KJ704274, KJ704281 – 82, 13: KJ704276 – 77, KJ704279, 14: KJ704278, KJ704280. Analyses were conducted in MEGA5 using the Jukes-Cantor model. All positions containing gaps and missing data were eliminated.

Comparisons between ATCC30254 and presumed *C. mellificae* sequences are highlighted in grey.
